# The gut microbiome and response to immune checkpoint inhibitors: preclinical and clinical strategies

**DOI:** 10.1186/s40169-019-0225-x

**Published:** 2019-03-18

**Authors:** Jun Gong, Alexander Chehrazi-Raffle, Veronica Placencio-Hickok, Michelle Guan, Andrew Hendifar, Ravi Salgia

**Affiliations:** 1 Department of Medicine, Division of Hematology/Oncology, Cedars-Sinai Medical Center, 8700 Beverly Blvd, Los Angeles, CA 90048 USA; 2 Department of Internal Medicine, Harbor-UCLA Medical Center, 1000 W Carson St, Torrance, CA 90509 USA; 3 Medical Oncology and Experimental Therapeutics, City of Hope Comprehensive Cancer Center, Building 51, Room 101, 1500 E Duarte St, Duarte, CA 91010 USA

**Keywords:** Gut microbiome, Commensal bacteria, Biomarkers, PD-1, PD-L1, CTLA-4, Immune checkpoint inhibitors

## Abstract

There is growing interest in identifying predictive biomarkers for inhibitors of programmed cell death protein 1 receptor (PD-1), programmed death ligand 1 (PD-L1), and cytotoxic T-lymphocyte associated protein 4 (CTLA-4). Given the links between the stool microbiota, anticancer immunosurveillance, and general health, the composition of the gut microbiome has recently undergone investigation as a biomarker for immunotherapy. In this review, we highlight published results from preclinical and clinical studies to date supporting a relationship between the gut microbiome and antitumor efficacy of immune checkpoint inhibitors. Despite the promising and hypothesis-generating findings that have been produced in this arena to date, there remain some inconsistencies amongst present data that may need to be resolved to contribute to further development. Among these, a better understanding of the immunomodulatory function of the microbiome, standardization in sampling, sequencing techniques, and data analysis, and ensuring uniformity across various aspects of study design are warranted in conducting future prospective studies seeking to validate the gut microbiome as a potential biomarker of response to checkpoint blockade.

## Introduction

There are currently several programmed cell death protein 1 receptor (PD-1) and programmed death ligand 1 (PD-L1) inhibitors approved by the Food and Drug Administration (FDA) in the treatment of solid and hematologic cancers [1]. As the clinical development of PD-1/PD-L1 inhibitors continues to pick up considerable momentum, so does the search for predictive biomarkers for this class of immunotherapy. Among the earliest and most widely recognized predictive biomarkers is PD-L1 expression though its absence in tumors certainly does not preclude response to PD-1/PD-L1 blockade [2].

Tumor mutational burden (TMB) has also been shown to predict benefit from immune checkpoint inhibitors across several tumor types due to generation of immunogenic neoantigens arising from an increased burden of nonsynonymous mutations [3]. Tumors harboring mutations in DNA mismatch repair genes resulting in microsatellite instability (MSI) or DNA polymerases (*POLE*) represent other phenotypes with high mutational load that can predict response to checkpoint blockade [4].

There is also growing interest in identifying the immune-active properties of the tumor microenvironment (TME) that constitute an immunologically “hot” tumor in responders to PD-1/PD-L1 blockade, in contrast to the immunologically “cold” tumor [5, 6]. For example, the type, density, and location of tumor infiltrating lymphocytes (TILs) are features that have been associated with response to checkpoint inhibition [7]. The Immunoscore represents a composite score incorporating such features of the infiltrating immune cell population and has been prospectively validated in colorectal cancer as a reliable prognostic indicator with further investigations in other tumors as a predictor of response to checkpoint blockade [8]. Profiling of the T-cell repertoire to assess for T-cell clonality may also serve as potential predictor of response to checkpoint inhibition [7]. Furthermore, assessment of a panel of markers associated with immune-sensitive or immune-resistant tumor phenotypes through gene expression profiling such as the Tumour Inflammation Signature or PanCancer IO 360 assay have shown promise in identifying candidates to PD-1/PD-L1 blockade [7].

More recently, the gut microbiome has emerged as another potential predictor of response to immune checkpoint inhibitors. The microbiome and its association with general health has long been described, and the potential to confer health benefits on the host through direct and indirect manipulation of the intestinal microflora has been a subject of investigation for the past several decades [9]. Examples of such manipulation have included probiotics, which are live microorganisms or biotherapeutic products that when administered in adequate amounts confer a health benefit on the host, and prebiotics, which are substrates such as carbohydrates or animal nutrition that are selectively used by host microorganisms to confer a potential health benefit. *Clostridium butyricum*, for instance, is a probiotic that has been shown to possess immunotherapeutic properties in cancer and gastrointestinal disorders [10, 11]. Prebiotics and synbiotics, which is a mixture of prebiotics and probiotics, have also demonstrated putative beneficial effects in the treatment of a multitude of other health conditions [12]. There are other microbiome studies investigating the relationship between the intestinal microbiota and efficacy of anticancer therapies, in general, in cancers of the lung and other organs, and the reader is referred to recent reviews [13–15]. In this review, we summarize the available evidence to date supporting the stool microbiota in shaping response to checkpoint blockade and their utility as a predictive biomarker for cancer immunotherapy. Beyond highlighting putative immunomodulatory mechanisms, we provide a phylogenetic classification of organisms associated with checkpoint inhibitor response and a succinct study-by-study tabulation of findings to allow one to readily compare results across preclinical and clinical studies. We also provide a novel discussion of inconsistencies across preclinical and clinical studies that does not serve to discredit the biomarker potential of the gut microbiome for checkpoint blockade, but rather to highlight areas in need of further investigation to strengthen the development of this exciting concept in immunotherapy.

## Preclinical studies

### CpG-oligonucleotides and anti-interleukin antibodies

An eloquent study involving mice subcutaneously injected with melanoma (B16) and colon carcinoma (MC38) cells pretreated with an antibiotic cocktail was among the first to show the relationship between the stool microbiome and response to immunotherapy [16]. Antibiotic-treated and germ-free mice showed significantly shorter survival and less tumor volume reduction with immunotherapy through injections of CpG-oligonucleotides and anti-interleukin (IL)-10 antibodies, when compared to controls, and highlighted that commensal gut microbiota primed tumor-infiltrating myeloid-derived cells through Toll-like receptor 4 (TLR4) activation and produce cytokines such as tumor necrosis factor (TNF) critical to antitumor efficacy (Table 1). Notably, administration of cultured *Allstipes* species (spp., *A. shahii*) or *Lactobacillus* spp. by gavage reconstituted or attenuated TNF-dependent tumor response to immunotherapy in antibiotic-treated mice, respectively (Table 1). Numbers of *Lactobacillus* spp. recovered as early as 1 week after stopping antibiotics, but recovery of *Allstipes* and *Ruminococcus* spp. was delayed, taking up to 4 weeks after stopping antibiotics.

**Table 1:** Published preclinical studies investigating the relationship between gut microbiota and antitumor efficacy of immunotherapy

Model	Microbial intervention	Immunotherapy	Findings	References
MC38 and B16 tumor-bearing mice	Vancomycin, imipenem/cilastatin, neomycin in drinking water Oral gavage	IT CpG-ODN and IP anti-IL-10 Abs	Impaired TNF-dependent antitumor activity and worse survival in antibiotic-treated and germ-free mice Number of *Allstipes* and *Ruminococcus* spp. positively correlated while *Lactobacillus* spp. negatively correlated with TNF-dependent tumor response to immunotherapy	[16]
MCA205, Ret, and MC38 tumor-bearing mice	Ampicillin, colistin, and streptomycin or imipenem Oral gavage Fecal transplantation	IP anti-CTLA-4 Ab	Impaired antitumor activity in germ-free or antibiotic-treated mice but not in specific pathogen-free mice In antibiotic-treated mice, oral feeding with *Bacteroides fragilis*, *Bacteroides thetaiotaomicron*, or *Burkholderia cepacia* recovered anti-CTLA-4 response In germ-free mice, oral feeding with *Bacteroides fragilis* recovered anti-CTLA-4 response Abundance of *Bacteroides* spp. (not *B. distasonis* or *B. uniformis*) correlated with response in human to mice fecal transplantation studies	[17]
B16.SIY and MB49 tumor-bearing mice	Oral gavage	IP anti-PD-L1 Ab	*Bifidobacterium* spp.-treated mice had significantly improved tumor control vs. non-*Bifidobacterium*-treated mice	[18]
BP tumor-bearing mice	Fecal transplantation	IP anti-PD-L1 Ab	Responders had significantly higher abundance of *Faecalibacterium* spp. and Ruminococcaceae family, while nonresponders had higher abundance of Bacteroidales order	[19]
MCA205, LLC, and Ret tumor-bearing mice	Ampicillin, colistin, and streptomycin Fecal transplantation Oral gavage	IP anti-PD-1 Ab ± anti-CTLA-4 Ab	Worse survival in antibiotic-treated and specific pathogen-free mice Reconstitution with *Akkermansia muciniphila* ± *Enterococcus hirae* or *Alistipes indistinctus* reversed resistance to PD-1 blockade in antibiotic-treated mice	[20]
B16.SIY tumor-bearing mice	Fecal transplantation	IP anti-PD-L1 Ab	2/3 mouse cohorts reconstituted with R fecal material showed slower baseline tumor growth 2/3 mouse cohorts reconstituted with NR fecal material showed faster baseline tumor growth Anti-PD-L1 efficacy seen in mice colonized with R microbiota vs. completely ineffective in NR-derived mice	[21]

*MC38* colon carcinoma, *B16* melanoma, *ODN* oligodeoxynucleotides, *IT* intratumoral, *IP* intraperitoneal, *IL-10* interleukin-10, *Abs* antibodies, *TNF* tumor necrosis factor, *spp.* species, *MCA205* sarcoma, *Ret* melanoma, *B16.SIY* melanoma, *CTLA-4* cytotoxic T-lymphocyte associated protein 4, *B16.SIY* melanoma, *MB49* bladder cancer, *PD-L1* programmed death ligand 1, *LLC* Lewis lung carcinoma, *PD-1* programmed cell death protein 1, *B16.SIY* melanoma, *R* responder, *NR* nonresponder

### Anti-CTLA-4 antibodies

In a subsequent study, tumor-bearing mice housed in germ-free conditions or treated with antibiotics experienced comprised antitumor effects with anti-cytotoxic T-lymphocyte associated protein 4 (CTLA-4) therapy that were associated with significantly decreased effector CD4+ T-cells and tumor-infiltrating lymphocytes (TILs), when compared to controls [17]. Oral feeding of these mice with various *Bacteroides* spp. or *Burkholderia* spp. restored response to anti-CTLA-4 therapy associated with T-helper 1 (TH_1_) immune responses in tumor-draining lymph nodes and maturation of intratumoral dendritic cells (DCs, Table 1). Fecal transplantation studies from metastatic melanoma patients to tumor-bearing, germ-free mice treated with anti-CTLA-4 therapy demonstrated abundance of *Bacteroides* spp. that correlated with response. Intestinal reconstitution of antibiotic-treated mice with *Bacteroides fragilis* and *Burkholderia cepacia* was also shown to reduce anti-CTLA-4-induced colitis.

### Anti-PD-L1 antibodies

In mice subcutaneously injected with melanoma and bladder cancer, response to anti-PD-L1 therapy was significantly correlated with *Bifidobacterium*-treated mice (oral gavage) compared to non-*Bifidobacterium*-treated mice that was associated with increases in interferon γ (IFN-γ)-producing tumor-antigen-specific T-cells, major histocompatibility complex (MHC) Class II dendritic cells, and upregulation of gene transcripts involved in CD8+ T-cell activation and costimulation, DC maturation, antigen processing and cross presentation, chemokine-mediated immune cell recruitment to the TME, and type I interferon signaling [18]. Of note, *Bifidobacterium* was not detected in mesenteric lymph nodes, spleen, or tumor suggesting that systemic antitumor immune responses occurred independently of bacterial translocation.

In a separate melanoma-bearing mouse model, response to anti-PD-L1 therapy significantly correlated with fecal transplantations from patients abundant in Ruminococcaceae family and *Faecalibacterium* spp., while nonresponders to PD-L1 blockade had abundance in stool Bacteroidales order (Table 1). Mice responsive to checkpoint inhibition had significantly higher levels of CD8+ TILs and TME PD-L1 expression but lower levels of CD11b+CD11c+ suppressive myeloid cells compared to nonresponders, while increases in RORγT+ Th17 tumor-infiltrating cells and regulatory CD4+ FoxP3+ T-cells and CD4+ IL-17+ T-cells were observed in nonresponders [19].

### Anti-PD-1 antibodies

In mice established with sarcoma and melanoma, 2 weeks of broad-spectrum antibiotics and rearing in specific pathogen-free conditions adversely affected survival with PD-1 ± CTLA-4 blockade [20]. Reconstitution with commensals such as *A. muciniphila* and *E. hirae* reversed resistance to PD-1 blockade in antibiotic-treated mice (Table 1). Interestingly, reconstitution with immune-sensitizing microbes was associated with accumulation of memory CCR9-expressing Th1-associated chemokine receptor-expressing CD4+ T-cells in tumor beds, metastatic lymph nodes, and draining lymph nodes 48 h after the first injection of anti-PD-1 antibody, formation of intratumoral granulomas, DC-induced IL-12 secretion, and increased CD4/Foxp3 ratios.

In a recent study involving fecal transplantation from melanoma patients who were responders and nonresponders to anti-PD-1 therapy into melanoma-bearing germ-free mice, anti-PD-L1 therapy was effective in mice colonized with responder microbiota and ineffective in mice colonized with nonresponder microbiota [21]. Responder microbiota-reconstituted mice had significantly higher numbers of SIY-specific CD8^+^ T cells, but not FoxP3^+^CD4^+^ regulatory T cells in the TME compared to nonresponder-derived mice.

## Clinical studies

### Baseline gut microbiome diversity

Numerous clinical studies investigating the stool microbiome in patients treated with checkpoint inhibitors have since been conducted in an attempt to corroborate findings demonstrated in preclinical models (Table 2). A prospective study collected buccal and fecal samples from 112 patients with metastatic melanoma prior to treatment with anti-PD-1 therapy [19]. Responders to anti-PD-1 therapy were significantly associated with higher diversity of gut microbiome and enriched with a unique stool bacterial composition compared to nonresponders; these findings were not observed in the oral microbiome (Table 2). Univariate analyses identified that the strongest predictors of response to anti-PD-1 therapy were alpha diversity [intermediate hazard ratio (HR) 3.60, 95% confidence interval (CI 1.02–12.74); abundance of *Faecalibacterium* genus (HR 2.92, 95% CI 1.08–7.89), and abundance of Bacteroidales order (HR 0.39, 95% CI 0.15–1.03)] in the gut microbiome. Interestingly, a significant positive correlation between tumor-infiltrating CD8+ TILs and higher levels of CD4+ and CD8+ T-cells in the systemic circulation with preserved cytokine response and abundance of the *Faecalibacterium* genus, Ruminococcaceae family, and Clostridiales order in the gut was observed. Conversely, a nonsignificant negative association between abundance of the Bacteroidales order and CD8+ TILs was observed. Higher abundance of Bacteroides order in the gut was associated with higher systemic levels of regulatory T-cells (Tregs) and myeloid derived suppressor cells (MDSCs) with a blunted cytokine response.

**Table 2:** Published clinical studies investigating the relationship between gut microbiota and antitumor efficacy of immunotherapy

Study	Tumor (n)	Checkpoint inhibitor	Findings	References
PS	Metastatic melanoma (n = 43)	Anti-PD-1 therapy (agent and dose not specified)	Higher diversity of gut microbiome in R (n = 30) vs. NR (13, p < 0.01), not observed in oral microbiome Enrichment of Clostridiales order/Ruminococcaceae family in R vs. enrichment of *Bacteroides thetaiotaomicron*, *Escherichia coli*, and *Anaerotruncus colihominis* in NR, not observed in oral microbiome Median PFS undefined with high vs. 242 days with low abundance of *F*aecalibacterium genus (p = 0.03); median PFS 188 days with high vs. median PFS 393 days with low abundance of Bacteroidales order (p = 0.05)	[19]
RS	Advanced NSCLC (n = 60), RCC (n = 40)	Anti-PD-1 therapy (agent and dose not specified)	*Akkermansia muciniphila* was significantly enriched in R vs. NR (validated in subsequent cohort with 27 NSCLC and 26 RCC pts) In the NSCLC cohort, *A. muciniphila* was also enriched in R (p = 0.045 with/without antibiotics, p = 0.026 excluding antibiotic-treated) along with *Ruminococcus* spp., *Alistipes* spp., *Eubacterium* spp., *Bifidobacterium adolescentis*, *Bifidobacterium longum*, and *Parabacteroides distasonis*	[20]
PS	Metastatic melanoma (n = 26)	I3 or 10 mg/kg Q3 weeks → maintenance Q12 weeks	In 7 pts with immune-related colitis, significant reductions from baseline to time of colitis seen in Firmicutes phylum (*Ruminococcus*, Lachnospiracea incertae sedis, *Blautia*, *Clostridium* IV, *Eubacterium*, unclassified Lachnospiraceae and Pseudoflavonifractor) and colitis associated with decreased bacterial diversity Baseline enrichment in *Faecalibacterium* genus and Firmicutes phylum (unclassified Ruminococcaceae, Clostridium XIVa and Blautia) significantly associated with longer PFS (p = 0.0039) and OS (p = 0.051) vs. *Bacteroides* spp. (independent of clinical characteristics) Baseline enrichment with Firmicutes phylum significantly associated with developing colitis (p = 0.009) vs. Bacteroidetes phylum in those who did not develop colitis (p = 0.011)	[24]
PS	Metastatic melanoma (n = 39)	I3 mg/kg Q3 weeks X4 doses, N 1 mg/kg + I3 mg/kg Q3 weeks X4 doses → N 240 mg Q2 weeks, N 240 mg Q2 weeks, or P 2 mg/kg Q3 weeks	In all pts: Baseline enrichment with *Bacteroides caccae* (p = 0.032 and *Streptococcus parasanguinis* (p = 0.048) in R vs. NR In N + I arm: Baseline enrichment with Firmicute phylum (*Faecalibacterium prausnitzii* (p = 0.032) and *Holdemania filiformis* (p = 0.043)) and Bacteroidetes phylum [*Bacteroides thetaiotamicron* (p = 0.046)] in R vs. NR In P arm, baseline enrichment with *Dorea formicigenerans* (p = 0.045) in R vs. NR In all pts, 83 gut metabolites at baseline were significantly different in R vs. NR (49 increased, 34 decreased, p < 0.05)	[25]
RS	Locally advanced or metastatic NSCLC (n = 15)	N 3 mg/kg Q2 weeks	73.3% received antibiotic monotherapy, 53.3% antibiotic duration > 7 days, 53.3% received antibiotics 1–3 months before first N, 33.4% < 1 month, and 13.3% during N Rate of CR 26.7%, SD 33.3%, PD 40% in antibiotic-treated vs. rate of CR 22%, SD 27.1%, PD 50.9% in non-antibiotic-treated (p = 0.75) No impact of antibiotics on PFS under N (p = 0.72)	[22]
RS	Advanced RCC (n = 16) or NSCLC (n = 48)	Anti-PD-1 or anti-PD-L1 antibody ± anti-CTLA-4 antibody (agents and dose not specified)	In RCC, PD rate 75% vs. 22% (p < 0.01), median PFS 1.9 vs. 7.4 months (HR 3.1, 95% CI 1.4–6.9, p < 0.01), median OS 17.3 vs. 30.6 months (HR 3.5, 95% CI 1.1–10.8, p = 0.03) in antibiotic-treated vs. no antibiotics (up to 30 days) In NSCLC, PD rate 52% vs. 43% (p = 0.26), median PFS 1.9 vs. 3.8 months (HR 1.5, 95% CI 1.0–2.2, p = 0.03), median OS 7.9 vs. 24.6 months (HR 4.4, 95% CI 2.6–7.7, p < 0.01) in antibiotic-treated vs. no antibiotics (up to 30 days)	[23]
RS	Metastatic melanoma (n = 42)	Anti-PD-1 or anti-CTLA-4 therapy (agent and dose not specified)	8 spp. more abundant at baseline in R: *Enterococcus faecium*, *Collinsella aerofaciens*, *Bifidobacterium adolescentis*, *Klebsiella pneumoniae*, *Veillonella parvula*, *Parabacteroides merdae*, *Lactobacillus* spp., and *Bifidobacterium longum* 2 spp. more abundant at baseline in NR: *Ruminococcus obeum* and *Roseburia intestinalis*	[21]

*PS* prospective study, *PD-1* programmed cell death protein 1 receptor, *R* responders per the response evaluation criteria in solid tumors (RECIST 1.1) criteria, *NR* nonresponders, *PFS* progression-free survival, *RS* retrospective study, *NSCLC* non-small cell lung cancer, *RCC* renal cell carcinoma, *pts* patients, *I* ipilimumab, *Q* every, *OS* overall survival, *N* nivolumab, *P* pembrolizumab, *CR* complete response, *SD* stable disease, *PD* progression disease, *HR* hazard ratio, *CI* confidence interval

A recent investigation collected baseline stool samples from 42 patients with metastatic melanoma prior to anti-PD-1 therapy [21]. After removing operational taxonomic units (OTUs) found in < 10% of samples and integration of 16S ribosomal RNA gene sequencing, metagenomic shotgun sequencing, and quantitative polymerase chain reaction (PCR), a selection of 10 spp. was produced with differential abundance in responders and nonresponders to PD-L1 blockade (Table 2). Fecal transplantation from responding and nonresponding patients into melanoma-inoculated mice treated with anti-PD-L1 therapy largely recapitulated outcomes and enrichment patterns seen in original donors.

### Effects of antibiotics

Clinical studies have also brought to attention the potential influence of antibiotics on outcomes in patients treated with checkpoint inhibitors. In one study of 249 patients with advanced non-small cell lung cancer (NSCLC, n = 140), renal cell carcinoma (RCC, n = 67), and urothelial carcinoma (n = 42) treated with PD-1/PD-L1 blockade after ≥ 1 prior therapies, treatment with antibiotics (beta-lactam inhibitors, fluoroquinolones, or macrolides) 2 months before or 1 month after PD-1/PD-L1 blockade was significantly associated with shorter progression-free survival (PFS) and overall survival (OS) [20]. Shotgun sequencing identified an overrepresentation of bacterial genera most notably including *Akkermansia muciniphila* in responders to PD-1 inhibition compared to nonresponders (Table 2, with or without antibiotics). Only Th1 and Tc1-cell reactivity against *A. muciniphila* and IFN-γ production above median were significantly associated with PFS in patients treated with PD-1 antibody. Oral gavage of sarcoma-carrying mice with stool samples from NSCLC patients who were responders and nonresponders recapitulated sensitivity and resistance to PD-1 blockade, respectively.

One retrospective study of patients with locally advanced or metastatic NSCLC investigated the outcome of patients treated with nivolumab in the setting of antibiotic exposure [22]. Out of 15 patients treated with antibiotics, response and PFS was not significantly different among those receiving nivolumab exposed and not exposed to antibiotics (Table 2). This study contradicts that of a larger retrospective study assessing the benefit of checkpoint blockade in advanced RCC and NSCLC patients exposed to antibiotics up to 30 or 60 days before the first dose of checkpoint inhibitor [23]. Increased rates of progressive disease (PD), shorter PFS, and shorter OS were observed in RCC patients exposed to antibiotics up to 30 days, and shorter PFS and OS were observed in NSCLC patients exposed to antibiotics up to 30 days (Table 2). Results were largely similar on analysis of RCC patients exposed to antibiotics up to 60 days before first dose of checkpoint inhibitor. Although antibiotic use and tumor burden were independently associated with worse PFS but not OS on multivariate analysis in the RCC cohort, antibiotic use was independently associated with worsened OS in the NSCLC cohort.

### Immune-mediated colitis

Clinical studies have also recently begun to describe the influence of the microbiota in modulating a unique toxicity of checkpoint blockade—immune-mediated colitis. In a prospective cohort of metastatic melanoma patients treated with ipilimumab, serial fecal samples were collected [24]. Relative reductions in gut microbiota were observed from baseline to time of onset of immune-related colitis in various members of Firmicutes phylum. Interestingly, baseline enrichment with Firmicutes phylum was significantly associated with developing colitis (p = 0.009) while significant enrichment in Bacteroidetes phylum was seen in those who did not develop colitis (p = 0.011). Patients who developed ipilimumab-induced colitis had significantly higher numbers of CD4+ T-cells but lower levels of IL-6, IL-8, and sCD25 at baseline compared to those without colitis. Notably, the investigators showed that antibiotics before ipilimumab treatment did not influence baseline dominant microbiota and none of the potentially predictive taxa were associated with antibiotic use.

### Baseline gut microbiota and metabolic signatures

A separate prospective cohort of 39 metastatic melanoma patients, of which 8% had used antibiotics prior to and/or during checkpoint blockade and 3% used probiotics, underwent metagenomic and metabolomic shotgun sequencing and provided a snapshot of baseline or pretreatment gut microbiota signatures associated with response to checkpoint inhibitors as well as significantly enriched and depleted metabolites involved in numerous metabolic pathways in responder metabolomes (Table 2) [25].

## Discussion

The list of potential biomarkers that predict response, or lack of through primary, adaptive, and acquired resistance, to checkpoint inhibitors is growing [26]. In the past 5 years, research into the association between the gut microbiome and response to PD-1/PD-L1/CTLA-4 inhibitors has produced interesting findings on the topic (Tables 1, 2). The list of microbes that have been positively correlated with response to checkpoint blockade in the preclinical realm include: *Bacteroides* spp. and *Burkholderia* spp. (anti-CTLA-4), *Bifidobacterium* spp., *Faecalibacterium* spp., and more broadly, Ruminococcaceae family (anti-PD-L1), and *Akkermansia muciniphila*, *Alistipes indistinctus* (of the Bacteroidales order), and *Enterococcus hirae* (anti-PD-1, Table 1). However, abundance of stool Bacteroidales order (includes *Bacteroides* spp.) has been associated with nonresponders to anti-PD-L1 therapy in a separate preclinical study [19].

**Fig. 1:**
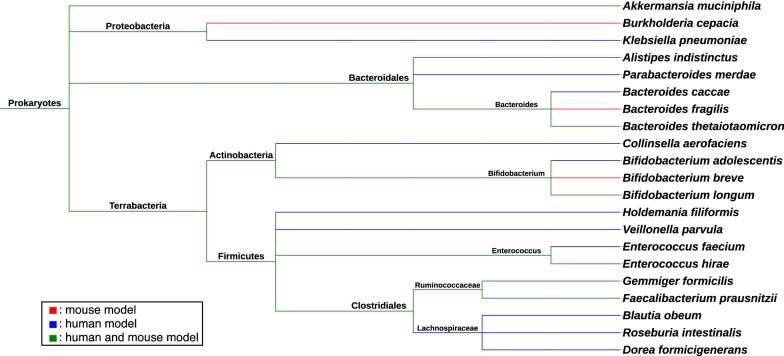
Phylogenetic tree of gut commensal bacteria associated with response to immune checkpoint inhibitors in both preclinical and clinical studies. Taxonomic classification is based on (from left to right) domain, phylum, order, family, genus, and species except for Terrabacteria (unranked). Figure created using the phylogenetic tree software by: [44]

In clinical studies, findings that are both concordant and discordant to other clinical and preclinical studies on the gut microbiome have been produced (Table 2). Enrichment in the Firmicutes phylum (includes the Clostridiales order, e.g., *Dorea formicigenerans*, *Eubacterium* spp., and *Veillonella parvula*, Ruminococcaceae family, e.g., *Ruminococcus* spp., *Blautia* genus, *Faecalibacterium* genus, e.g., *Faecalibacterium prausnitzii*, and individual organisms *Enterococcus faecium*, *Holdemania filiformis*, *Lactobacillus* spp., and *Streptococcus parasanguinis*), *Bifidobacterium adolescentis*, *Bifidobacterium longum*, *Akkermansia muciniphila*, *Collinsella aerofacien*, *Klebsiella pneumoniae*, *Alistipes* spp. (of the Bacteroidales order), and *Parabacteroides merdae*/*distasonis* (of the Bacteroidales order) have been associated with response to PD-1 and CTLA-4 blockade in humans (Fig. 1) [19–21, 24, 25], while Bacteroidales order (includes *Bacteroides* spp., e.g., *Bacteroides thetaiotaomicron*), *Escherichia coli*, and *Anaerotruncus colihominis* (of the Clostridiales order/Ruminococcaceae family), and *Roseburia intestinalis* (of the Clostridiales order) have been negatively associated with response to anti-PD-1 and anti-CTLA-4 therapy [19, 21, 24]. Notably, baseline enrichment in Bacteroidetes phylum (includes *Bacteroides thetaiotamicron* and *Bacteroides caccae*) has been associated with response to anti-PD-1 and anti-CTLA-4 therapy in melanoma patients [25], which is in contrast to some preclinical and clinical evidence described previously that support their abundance as associated with lack of response. Furthermore, lack of response to anti-PD-1 or anti-CTLA-4 therapy in another melanoma cohort has been associated with baseline abundance in *Ruminococcus obeum*, which contradicts other preclinical/clinical data supporting that gut enrichment with Ruminococcaceae family and *Ruminococcus* spp. positively correlate with response to checkpoint inhibitors [21].

Where the utility of the stool microbiota falls along the spectrum of clinically-relevant biomarkers for checkpoint blockade is unclear given the incongruent findings present in both published preclinical and clinical studies to date. Although interesting and thought-provoking, there remain a number of critical issues at hand that need to be addressed in order to establish the candidacy of the gut microbiome as a predictive biomarker for this promising class of immunotherapy.

### Immunomodulatory mechanisms

**Fig. 2:**
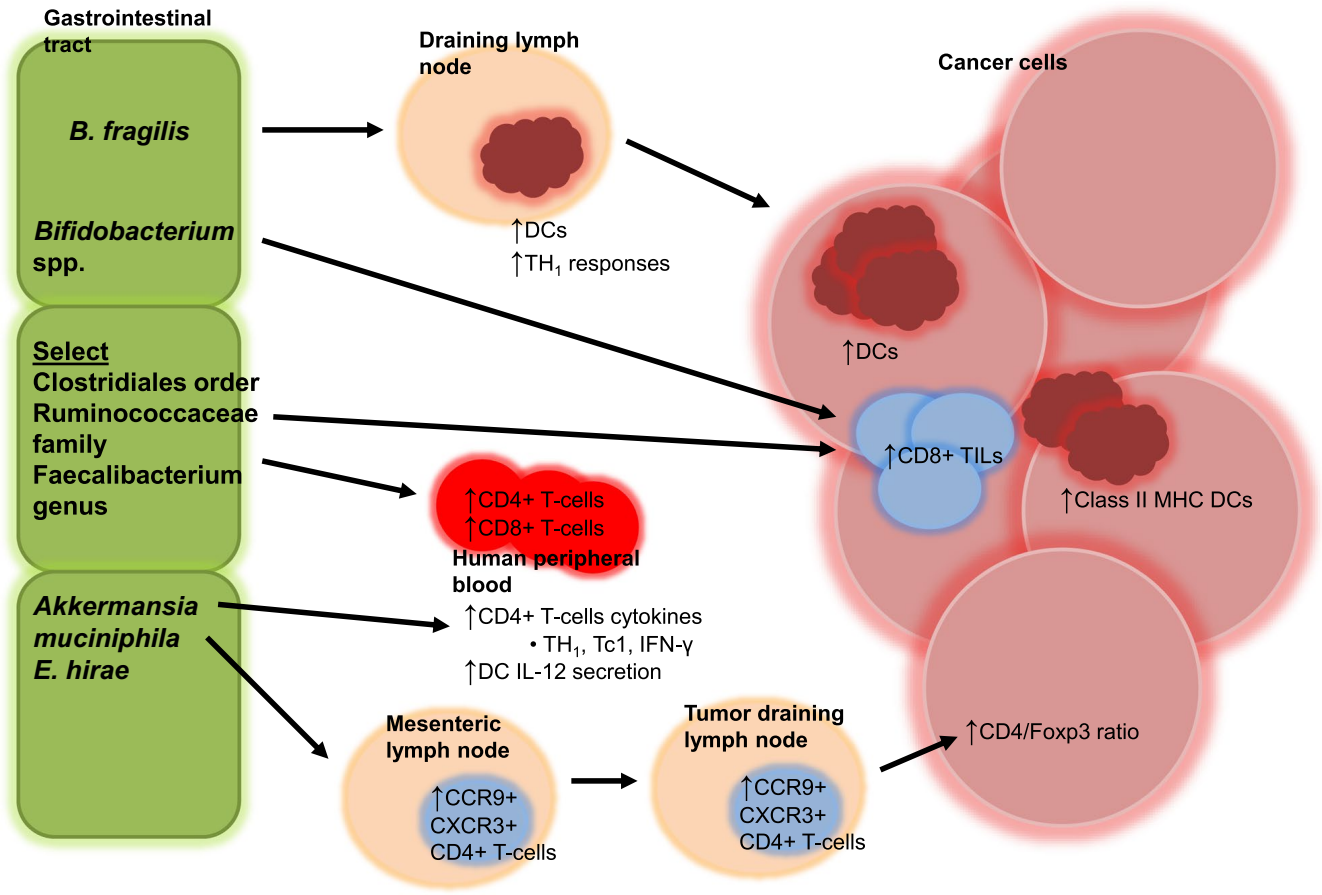
Proposed immunomodulatory mechanisms of commensal bacteria on anticancer efficacy of immune checkpoint inhibitors in animal models and patients. Oral gavage of B. fragilis in germ-free mice has been shown to induce T helper 1 (TH_1_) immune responses in tumor-draining lymph nodes and maturation of dendritic cells (DCs) in responders to cytotoxic T-lymphocyte associated protein 4 (CTLA-4) blockade. Oral gavage of Bifidobacterium spp. in mice was shown to increase accumulation of antigen-specific CD8^+^ tumor-infiltrating lymphocytes (TILs) and major histocompatibility complex (MHC) Class II DCs in responders to programmed death ligand 1 (PD-L1) blockade. Human responders to programmed cell death protein 1 receptor (PD-1) blockade had significant positive correlations between CD8+ TILs or levels of CD4+ and CD8+ T-cells in the peripheral blood and abundance of select members of the Clostridiales order, Ruminococcaceae family, and *Faecalibacterium* genus. Oral gavage of *Akkermansia muciniphila* and *E. hirae* was associated with increased central memory CD4+ T-cells expressing the small intestine-associated chemokine receptor CCR9 and/or the TH_1_-associated chemokine receptor CXCR3 in mesenteric and tumor draining lymph nodes as well as increased CD4/Foxp3 ratios in tumors of mice cotreated with anti-PD-1 therapy. In human peripheral blood, secretion of cytokines by CD4+ T-cells including TH_1_, Tc1, and interferon-γ (IFN-γ) and bone marrow-derived DCs including IL-12 were associated with response to PD-1 blockade and reactivity against *A. muciniphila* and *E. hirae* (for Tc1)

It has long been implicated that the microbiome is involved in tumorigenesis as well as activation or suppression of the immune system that can contribute to tumor control or escape [27, 28]. Early attempts in linking the gut microbiome and anticancer immunosurveillance hypothesized that (1) microbial antigens may sufficiently stimulate antitumor immune activity through tumor antigenic mimicry or cross-reactivity, (2) microbes may provide a non-antigenic co-stimulus or secondary signal (or collection of signals) resulting in bystander activation of tumor associated antigen-specific T lymphocytes, and/or (3) microbial toxins and byproducts may directly or indirectly (through immunosurveillance) affect cancer cells [28]. Specific to the antitumor activity of immune checkpoint inhibitors, a growing body of evidence now posits that the gut microbiota may enhance the function of DCs with more potent tumor antigen presentation and cytokine production, increase trafficking of CD4+ memory T-cells from mesenteric and draining lymph nodes to the TME, decrease Tregs and MDSCs, and increase recruitment and activation of IFN-γ-producing tumor-antigen-specific effector T-cells that altogether contribute to the modulation of the antitumor immune response (Fig. 2) [29].

Evidence is also accumulating to support that immunoregulatory pathways that facilitate checkpoint inhibitor response may be commensal-specific [30]. In preclinical models, inoculation of mice with *B. fragilis*, *A. muciniphila*, and *E. hirae* have been shown to induce TH_1_ immune responses, promote maturation of DCs, and increase central memory CD4+ T-cells in mesenteric lymph nodes, tumor draining lymph nodes, and/or the TME in response to checkpoint inhibitors [17, 20]. Oral gavage of *Bifidobacterium* spp. in mice cotreated with anti-PD-L1 therapy was shown to increase antigen-specific CD8+ TILs and MHC Class II DCs, while abundance of Clostridiales order, Ruminococcaceae family, and *Faecalibacterium* genus was associated with increased CD8+ TILs and peripheral blood CD4+/CD8+ T-cells in human responders to PD-1 blockade [18, 19]. Abundance of *A. muciniphila* and *E. hirae* has been shown to be associated with secretion of cytokines by MCH Class II-restricted CD4+ T-cells and DCs in the peripheral blood of human responders to PD-1 blockade (Fig. 2) [20].

Despite the initial insights into the immunomodulatory mechanisms of the stool microbiome, the exact mechanisms linking commensal bacterial species to the anticancer efficacy of checkpoint blockade in animal models and humans remain elusive. Our understanding of the impact of gut commensals on checkpoint inhibitor response has benefited greatly from experiments performing immune profiling in subjects treated with immunotherapy and inoculated with specific bacteria [17–20]. Further insights into direct cause-effect relationships between checkpoint inhibitor response and stool microbiota have been afforded by fecal transplantation from human responders of immunotherapy to mice with in-depth characterization of immune responses [19–21]. However, these studies did not further identify the specific bacteria whose abundance was associated with immune responses; in recognition that fecal transplantation from human responders can contain a diversity of microbials and that mechanisms of checkpoint inhibitor response can be commensal-specific, broader investigation involving inoculation with single-lineage bacteria and immune profiling in responders would be prudent in our understanding of gut microbiome-facilitated response to immunotherapy.

An overarching question in this area is whether the abundance of stool bacteria associated with response to checkpoint blockade is simply a reflection of the presence of health-associated bacteria that are of usual higher quantities in healthier individuals with more robust and functional immune systems or is it through mechanisms of the bacteria themselves that determine the host immune system’s capability to engage in antitumor responses [30]. On this latter note, it should also be asked whether the antitumor immune response is dependent solely on bacterial properties and their direct interactions with the immune checkpoint inhibitors or through interactions involving the host-bacterial ecosystem and immunomodulatory cells [31]. Another research strategy to improve our understanding in this arena could entail investigating the magnitude by which gut commensals themselves stimulate innate and adaptive antitumor immune responses; these analyses have been initially presented in several studies [17, 18, 20]. Future study in controlled experiments evaluating immune profiles from inoculation of stool microbiota with and without checkpoint inhibitors could provide further understanding of (1) whether immune response pathways elicited by commensals are distinct from those generated by checkpoint blockade in altogether providing synergistic antitumor activity or (2) whether checkpoint blockade elicits antitumor responses that overlap the same immune response pathways activated in recognition of bacterial antigens and byproducts. Additionally, greater understanding of underlying mechanisms may be afforded in research on the contribution of the microbiome to therapy-induced anticancer immune responses across other treatment modalities beyond checkpoint inhibition such as chemotherapy, radiation therapy, hematopoietic stem cell transplantation, and other forms of immunotherapy [32, 33].

Furthermore, metabolomics analysis has recently identified significant differences in 83 gut metabolites at baseline in responders to anti-PD-1 and anti-CTLA-4 therapy compared to nonresponders with metastatic melanoma [25]. In essence, bacterial metabolites and byproducts of metabolic pathways involved in amino acid metabolism, lipid metabolism, nucleotide metabolism, and carbohydrate metabolism may also affect response to checkpoint blockade. As the putative mechanisms by which commensal bacteria facilitate response to immunotherapy increases in complexity, further understanding of the relationships between the gut microbiome and the antitumor immune response is critical in predicting success to checkpoint blockade.

### Translation from preclinical to clinical settings

As stated previously, several inconsistencies in the gut microbiome composition have been produced in recent preclinical and clinical studies focused on investigating the relationship between stool microbiota and response to checkpoint inhibition (Tables 1, 2). Beyond associations between specific commensals and response (or lack of) to checkpoint blockade, increased representation of baseline Bacteroidetes phylum (includes *Bacteroides fragilis*) in melanoma patients and intestinal reconstitution with *Burkholderia cepacia* in antibiotic-treated, tumor-bearing mice have been shown to reduce anti-CTLA-4-induced colitis potentially by limiting inflammation through stimulation of Treg differentiation [17, 24, 34]. This is in contrast to studies showing an association with colonization by *Bacteroides* spp. and ulcerative colitis and Crohn’s disease in mice models and humans [35–38]. Moreover, antibiotic use has been correlated with poorer outcome in tumor-carrying mice and metastatic RCC and NSCLC patients treated with anti-PD-1 and anti-CTLA-4 antibodies [17, 20, 23]. However, in 1 prospective cohort of metastatic melanoma patients treated with ipilimumab and 1 retrospective cohort of advanced NSCLC patients treated with nivolumab, antibiotic use had no impact on response to checkpoint blockade or association on potentially predictive taxa [22, 24]. Lastly, a higher diversity of the gut microbiome in responding patients with melanoma to anti-PD-1 therapy was observed compared to nonresponders [19]. However, a separate melanoma cohort identified that there were no significant differences in the level of gut microbial diversity between responders and nonresponders to anti-PD-1 and anti-CTLA-4 therapy [25].

These inconsistencies across preclinical and clinical studies highlight several important points that need to be considered in development of future research in this area. Firstly, caution should be taken in extrapolating data from mice studies into humans. The anatomical structures and intestinal wall linings have been shown to significantly differ across human and mouse gastrointestinal tracts [39]. It has also been shown that 85% of the bacterial genera found in the mouse gut microbiome is not present in humans [40]. Furthermore, dynamic shifts in microbial species distribution can often occur due to host diet or lifestyle as well as interspecies competitive exclusion [31, 41]. Sampling and sequencing technique of stool specimens is another factor that can introduce variability in correlating the composition of the gut microbiome with checkpoint inhibitor response. Most human gut microbiome studies utilize stool samples, while mouse gut microbiome studies usually rely on cecal contents unless pellets are sampled in some longitudinal studies [39]. Historically, the standard choice for mouse studies has been mostly 16S rRNA sequencing whereas human microbiome studies have used both metagenomic and 16S rRNA sequencing approaches [39]. Metagenomic shotgun sequencing has several potential advantages over 16S rRNA sequencing as it can eliminate PCR bias seen with taxa that are over- or underrepresented depending on the choice of primers and 16S rRNA variable region to be amplified, improve gut microbiome taxonomic resolution at the species level given that bacteria belonging to the same genus can have different phenotypes or host effects, and provide information on metabolic pathways of the microbiome [25]. Nevertheless, variability can exist in either strategy due to differences in collection, storage, and processing of stool samples, extraction protocols for nucleic acids, and approaches used in data analysis [30].

A third consideration encompasses study design, which has general applicability across models despite its particular relevance to non-preclinical studies. Differences in study design including retrospective vs. prospective design, sample size, experimental subject and tumor heterogeneity, and checkpoint inhibitor such as anti-CTLA-4 vs. anti-PD-1/PD-L1 can certainly account for the variability in findings across microbiome studies in animals and humans [31]. Differences in frequency of sampling can also affect the accuracy to describe variations in taxome distribution over time given that although the individual gut microbiome can remain stable for long durations of time, changes in composition of the microbiome can rapidly occur due to antibiotics, dietary, and environmental changes [25]. In the largest cohort to date investigating the impact of antibiotics on the gut microbiota and response to checkpoint blockade, factors with potential impact on the microbiota composition such as diet, country of origin, and use of other medications were not taken into account [23]. It should be pointed out that although a detrimental effect of antibiotics on response to checkpoint blockade was identified in this study, the authors are unclear whether this reflects a general prognostic association or a causative link with resistance to checkpoint inhibition [23].

### Future directions for clinical studies

The gold standard in designing the ideal investigation of the gut microbiome composition as a predictor of response to checkpoint blockade would involve taking into consideration all of the above points and incorporating them into a study of large sample size and prospective design. This is easier said than done, but to ensure our success in conducting high-quality research with minimal bias and confounding factors in this arena, future efforts can implement several key study parameters. Techniques in sampling and sequencing should be standardized; in the case of 16S rRNA sequencing, it will be important to minimize variations in the many proposed algorithms for clustering of genetic sequences into OTUs to measure microbiome diversity that have been found to have a negative influence on downstream analyses [31]. Furthermore, serial and longitudinal sampling will be of value to assess changes in an individual’s gut microbiome over time in relation to checkpoint inhibitor response [25, 42]. To the best of our ability, controlling for or taking into account baseline differences in an individual’s microbiome profile across patient demographics such as sex, age, race, comorbidities, medications including antibiotics and probiotics, diet and lifestyle, and environment/geographic location will add greatly to the development of a more standard measurement for future microbiome investigations [31].

It is increasingly understood that the diversity of the gut microbiome may include some bacterial species that are immunosuppressive while others that are immune-stimulatory [43]. Rather than risk the likelihood of underestimating the total number of bacteria showing differential abundance in responders compared to nonresponders of checkpoint inhibition (a problem often encountered in 16S rRNA sequencing given that the analysis is limited by the number of samples above the detection threshold), representing the data in aggregate through construction of a ratio comprised of the total number of “beneficial” and “nonbeneficial” OTUs has demonstrated feasibility in producing a composite commensal microbiota score that is predictive of benefit to checkpoint blockade [21]. Furthermore, improvements in the isolation of cultivable bacteria and derivation of individual clones with implementation of whole-genome sequencing may represent future steps in our ability to study the composition of the gut microbiome [30]. In developing the ideal biomarker for checkpoint inhibitors beyond the gut microbiome, future investigations may expand their attention beyond bacteria to the broader ecological community such as viruses and fungi; integration of the microbiome with metabolomics, proteomics, and genomics may provide an even more comprehensive prognostic and predictive biomarker [30, 42].

Lastly, with better uniformity across sampling techniques, data analysis, and study design and a greater understanding of the immunomodulatory mechanisms of the microbiome, we will be primed to investigate strategies to modify the gut microbiome and potentially improve cancer outcomes. There are numerous ongoing clinical studies and prospective trials investigating the role of intestinal commensals and their effect on anticancer therapies (Table 3). Ideally, these studies will provide some clarity to many of the questions that have emerged on manipulation of the stool microbiome and cancer immunotherapy. In line with the concept of precision oncology, a future goal would involve manipulation of an individual’s microbiome through potential strategies including fecal microbial transplantation, provision of single bacterial species or a cocktail of beneficial organisms, dietary interventions, antibiotics, and/or probiotics to enhance the effect of anticancer therapies [30].

**Table 3:** Ongoing select clinical studies investigating the effect of gut microbiota on anticancer therapies

Study	Tumor, setting	Interventions	Primary endpoint(s)	NCT
Observational, n = 49	TNBC, newly diagnosed	Neoadjuvant chemotherapy with collection with pre- and post-therapy stool and PB samples	pCR rate as associated with composition of intestinal microbiota and subsequent short-term alterations in composition	NCT03586297
Observational, n = 80	Metastatic CRC, first-line; metastatic carcinoma, first-line anti-PD-1/PD-L1 therapy	FOLFOX or FOLFIRI or anti-PD-1/PD-L1 therapy with collection of pre-therapy and interval stool samples	Tumor response correlated with presence and amounts of species	NCT02960282
Observational, n = 120	AML, newly diagnosed or undergoing HSCT	Serial stool samples analyzed by next-generation sequencing	Association between changes in the intestinal microbiota and the incidence of gastrointestinal GVHD	NCT03148197
Case–control, n = 200	Glioblastoma multiforme, first-line	Concurrent chemoradiation (temozolomide) or radiation therapy or healthy control and collection of pre- and post-surgery stool samples	Pre-operative gut microbiome composition, perturbation of gut microbiota by temozolomide, and correlation of gut microbiota and prognosis	NCT03631823
Phase I, n = 40	Advanced melanoma, treatment refractory	FMT from responders of immunotherapy	Safety and comparison of gut microbiome composition pre- and post-FMT	NCT03353402
Phase I/II, n = 20	AML or high-risk MDS, first-line	Induction therapy + autologous FMT	Efficacy in dysbiosis correction by measure of microbiota diversity and eradication of MDRB	NCT02928523
Phase II, n = 20	Advanced melanoma, treatment refractory	FMT + pembrolizumab	ORR	NCT03341143
Phase II, n = 144	Any hematologic malignancy undergoing HSCT	Piperacillin–tazobactam or cefepime	Fold-change in Clostridiales abundance	NCT03078010

*NCT* ClinicalTrials.gov identifier, *TNBC* triple-negative breast cancer, *PB* peripheral blood, *CRC* colorectal cancer, *PD-L1* programmed death ligand 1, *PD-1* programmed cell death protein 1, *FOLFOX* 5-fluorouracil, leucovorin, and oxaliplatin, *FOLFIRI* 5-fluorouracil, leucovorin, and irinotecan, *AML* acute myeloid leukemia, *HSCT* hematopoietic stem cell transplantation, *GVHD* graft-versus-host disease, *FMT* fecal microbiota transplantation, *MDS* myelodysplastic syndrome, *MDRB* multidrug resistant bacteria, *ORR* overall response rate

## Conclusion

Preclinical and clinical evidence is accumulating to support an association between the gut microbiome composition and antitumor efficacy of immune checkpoint inhibitors. However, to further its advancement as a potential biomarker for immunotherapy, there are several inconsistencies amongst present data that should be addressed. A greater understanding of the immunomodulatory mechanisms of the microbiome, standardization of sampling, sequencing techniques, and data analysis, and ensuring uniformity in study design are key considerations that may need to be incorporated into future investigations. Ultimately, validation of findings from existing preclinical and clinical data in subsequent studies of large sample size and prospective design is warranted to further develop the stool microbiota as a biomarker for checkpoint blockade.

## Abbreviations

PD-1: programmed cell death 1
PD-L1: programmed death-ligand 1
CTLA-4: cytotoxic T-lymphocyte antigen 4
FDA: Food and Drug Administration
TMB: tumor mutational burden
MSI: microsatellite instability
POLE: DNA polymerase epsilon
TME: tumor microenvironment
TILs: tumor-infiltrating lymphocytes
B16: melanoma cell line
MC38: colon carcinoma cell line
IL: interleukin
TLR4: Toll-like receptor 4
TNF: tumor necrosis factor
spp.: species
TH_1_: T-helper 1
DCs: dendritic cells
IFN-γ: in interferon γ
MHC: major histocompatibility complex
HR: hazard ratio
CI: confidence interval
Tregs: regulatory T-cells
MDSCs: myeloid derived suppressor cells
OTUs: operational taxonomic units (OTUs)
PCR: polymerase chain reaction
NSCLC: non-small cell lung cancer
RCC: renal cell carcinoma
PFS: progression-free survival
OS: overall survival
PD: progressive disease
